# Single-Cell Profiling Identifies JUNB/SPI1-Driven Inflammatory Programs and Novel Communication Axes in Myeloid Cells of Sepsis

**DOI:** 10.2174/0118715303425071250925075308

**Published:** 2025-10-16

**Authors:** Liyao Liu, Lin Zhao, Jixiang Tan

**Affiliations:** 1 Department of Emergency, The First Affiliated Hospital of Chongqing Medical University, Chongqing, 400016, China

**Keywords:** Sepsis, single cell RNA-seq, myeloid cells, GSEA, inflammation, cell communication

## Abstract

**Introduction:**

Sepsis is a Systemic Inflammatory Response (SIR) caused by invading pathogens. We aimed to characterize infiltrating cells in sepsis and provide novel insight for the treatment of sepsis.

**Materials and Methods:**

Whole-blood scRNA-seq samples from four septic patients and five healthy subjects were collected from the Gene Expression Omnibus (GEO) database (GSE175453). The Seurat R package was used for quality control and cell clustering by scRNA-seq analysis. Gene set enrichment analysis (GSEA) was performed using the clusterProfiler R package for pathway enrichment analysis. Then, the SCENIC analysis was used to identify key transcriptional regulons, and the CellChat R package was used for cell communication analysis.

**Results:**

We mainly obtained 9 cell clusters, including myeloid cells, T cells, dendritic cells, NK T cells, B cells, plasma B cells, megakaryocytes, mast cells and erythrocytes. Notably, myeloid cells, erythrocytes and mast cells had a higher proportion in sepsis patients. Activated IL-17 and p53 pathways supported anti-infection response in myeloid cells, and *JUNB* and *SPI1* mediated multiple inflammatory pathways, including TNF signaling and neutrophil activation. We also identified that the cell interaction mode of myeloid cells, such as MPZL1-MPZL1 and FASL-FAS, may serve as a potential target for an anti-inflammatory response in sepsis treatment.

**Discussions:**

The scRNA-seq analysis revealed pro-inflammatory pathways (IL-17, p53) and key regulators (*JUNB*, *SPI1*) in septic myeloid cells. Receptor genes (MPZL1 and FAS) mediated cell communication, offering potential biomarkers and targets for sepsis therapy.

**Conclusion:**

We characterized the pro-inflammatory immune response pathways, transcriptional regulon and cell interaction modes of myeloid cells in the development of sepsis.

## 1 INTRODUCTION

Sepsis is a condition stemming from the host’s maladaptive response to infection, where most patients progress to multi-organ dysfunction and failure, sometimes with fatal consequences [1-3]. Sepsis manifests as an elevated pulse rate, an abnormal white blood cell count, a higher respiratory rate, and a drop in blood pressure, which reduces tissue perfusion pressure and impairs venous return, leading to septic cardiomyopathy and hypoxia [4]. At present, sepsis remains a major cause of global mortality and morbidity worldwide, with an estimated annual incidence of 30 million cases and 6 million deaths [5]. However, studies suggested that up to two-thirds of sepsis-related deaths may be preventable with proper clinical management [6], including early diagnosis, haemodynamic resuscitation and antimicrobial therapy [7]. Early detection and management of sepsis can be challenging, partly due to the difficulty in clearly distinguishing between severe infection with clinical suspicion and true sepsis. The complexity is further aggravated by the fact that opportunistic pathogens may originate not only from patients themselves but also from hospital environments, which have been shown to harbor diverse actinomycete species capable of surviving under harsh conditions and posing significant risks for cross-transmission to patients [8].

Additionally, sepsis has a highly complex pathomechanism that can manifest through certain nonspecific symptoms, including upper respiratory complaints, diaphoresis, or flushed skin. In pathophysiology, infectious pathogens are recognized by resident macrophages, specialized cells associated with phagocytosis; the process of detecting and destroying pathogens, to initiate a localized inflammatory response through various cytokines. Vascular lining receptors detect infective agents of the pathogen cell wall and recruit local infiltration of leukocytes, macrophages and neutrophils, among which neutrophils are the first specialist white blood cells migrating to the infection site to destroy micro-organisms [9]. Meanwhile, neutropenic sepsis represents an overwhelming infection for patients with a low neutrophil count, while the increased proliferation of white blood cells caused by sepsis is known as leukocytosis [10]. Although increasing white blood cells attempt to eliminate invasive pathogens, they can also damage the vascular lining cell (endothelium), leading to enhanced vascular permeability, causing capillary leakage, and producing excessive cytokines, interleukins, and Nitric Oxide (NO), which in turn contribute to the development of sepsis [11].

These circulating immune cells are crucial factors that cause persistent immunosuppression, inflammation, and catabolism syndrome [12]. Previous studies also revealed that the dismal long-term outcomes of sepsis patients are partly due to a failure of pre-sepsis immune status recovery of the host [13]. Understanding the pathobiology of infiltrating immune cells in sepsis and the resulting dysfunctional hematopoiesis that leads to immune dyscrasia is essential for successful immunomodulatory strategies. In this study, we performed Single Cell RNA-sequencing (scRNA-seq) analysis to characterize the unique immune niche of these circulating immune cells from the peripheral blood of sepsis patients. Pathway enrichment difference, crucial transcriptional regulons and cell communication of myeloid cells in sepsis and healthy patients were analyzed. We found that the activated interleukin-17 (IL-17) and p53 pathways mediated an anti-infection response; the Transcription Factors (TFs) JUNB and SPI1 regulated multiple inflammatory pathways. Common cell communication through MPZL1-MPZL1 and FASL-FAS in myeloid cells plays a vital role in sepsis development.

## 2 MATERIALS AND METHODS

### 2.1 Data Acquisition

We downloaded GSE175453 dataset from the Gene Expression Omnibus (GEO; https://www.ncbi.nlm.nih.gov/geo/) database and selected samples of sepsis and healthy controls based on the files provided for each cell corresponding to the sample information [14]. A total of 4 sepsis samples and 5 healthy control samples were obtained. The scRNA-seq analysis was performed using the 10x Genomics library building platform and the Illumina NovaSeq 6000 and Illumina HiSeq™ NextSeq 500 sequencing instruments.

### 2.2 Preprocessing of the scRNA-seq Data

We used the “Seurat” R package for quality control and processing of the scRNA-seq data [15, 16]. The Read10X function was used to read the expression matrix, retaining cells with a gene number of 200~6000 genes that were expressed by at least 3 cells with less than 10% mitochondrial genes. Highly variable genes were set to 3,000 and normalized by the SCTransform function. After performing Principal Component Analysis (PCA) with the RunPCA function, the “harmony” package was used to remove batch effects between different samples. Then, the top 20 principal components were used for dimensionality reduction by UMAP, and the cells were clustered using the “FindNeighbors” and “FindClusters” functions (resolution = 0.1) [17]. Finally, the marker genes from the CellMarker2.0 database (http://bio-bigdata.hrbmu.edu.cn/CellMarker/) were used for cell type annotation, and the subtype detection of myeloid cells was conducted using the same method described above.

### 2.3 Gene Set Enrichment Analysis (GSEA)

The FindMarkers function was used to calculate the gene-level log_2_FC value of cells in the sepsis group compared to those in the normal group. The enrichGO and enrichKEGG functions of the “clusterProfiler” R package were used for Gene Ontology (GO) in Biological Process (BP) terms and the Kyoto Encyclopedia of Genes and Genomes (KEGG) [18, 19]. Then, Differentially Expressed Genes (DEGs) were visualized by the “GseaVis” R package.

### 2.4 SCENIC Analysis

Transcriptional state differences drive cellular heterogeneity and are regulated by Gene Regulatory Networks (GRNs), which are dominated by TFs. SCENIC is an algorithm for GRN analysis of single-cell data, introducing gene co-expression networks inferred by the TF motif sequence validation method to improve the reliability of GRN identification. Based on the official tutorial of SCENIC, the GENIE3 method was used to identify potential target genes of each TF, and the top10perTarget method was employed to build a TF-mediated regulatory network, including high-confidence TF-target gene pairs (regulons) [20]. In addition, the AUCell function was used to assess the activity of regulons in each cell, and these regulon activities were scored by the “SCENIC” R package based on gene expression, with higher scores indicating stronger activity and importance.

### 2.5 Cell Communication Analysis

The receptor-ligand pairs were mapped using the “CellChat” R package [21]. Firstly, the createCellChat function was used to create a CellChat object, and the identifyOverExpressedGenes and identifyOverExpressedInteractions functions were used to identify the significant expression of receptor-ligand pairs among cell subgroups. Then, the projectData function was used for mapping the protein interaction network, and the computeCommunProb function was utilized to infer the probability of ligand-receptor pair interactions between cell subpopulations. Meanwhile, the probability of each ligand-receptor pair’s pathway was quantified by the computeCommunProbPathway function. Lastly, the bubble plot was created by the netVisual_bubble function.

### 2.6 Statistical Analysis

We used the R software (version 4.0.5) for data analysis and visualization, and the independent t-test was conducted for significant difference analysis on two continuous variables. A *p*<0.05 was considered statistically significant.

## 3 RESULTS

### 3.1 The Single-cell Landscape in Normal and Sepsis Samples

**Fig. (1):**
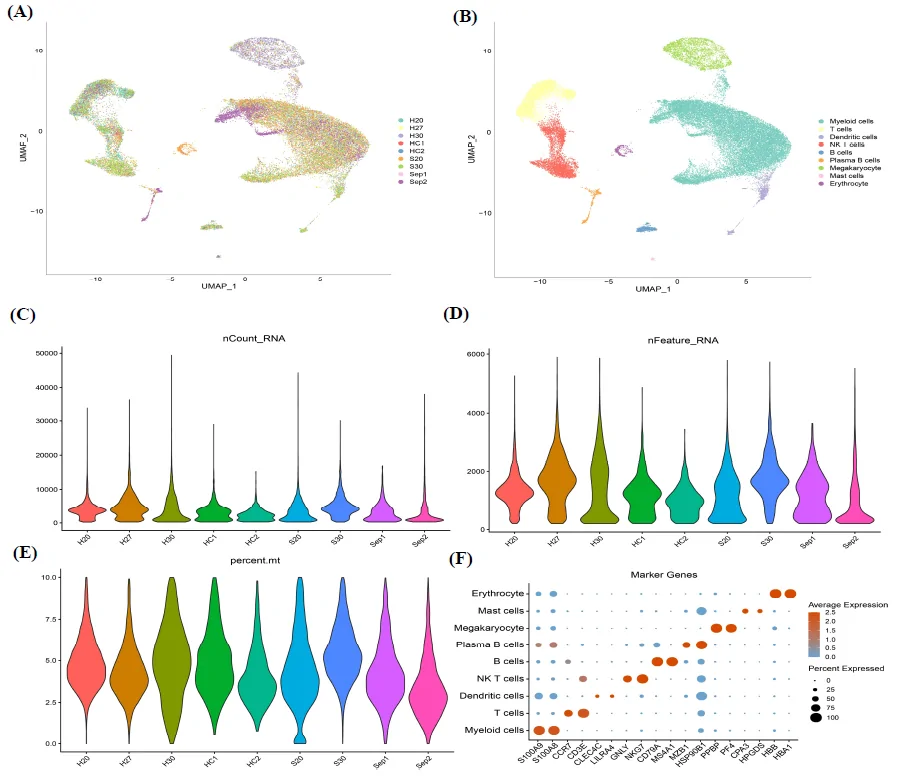
Single-cell landscape in sepsis and control samples. (**A**) UMAP plot of cell clustering in different samples. (**B**) UMAP of the annotated cell clustering. (**C**) The number of genes in each sample (X axis represents the gene number and the Y axis represents the different samples). (**D**) The number of genes in each cell cluster. (**E**) Violin plot of mitochondrial gene proportion in each cell cluster. (**F**) Bubble plot of marker gene expression in each cell cluster (X axis is the name of marker genes and Y axis is the different cell types).

**Fig. (2):**
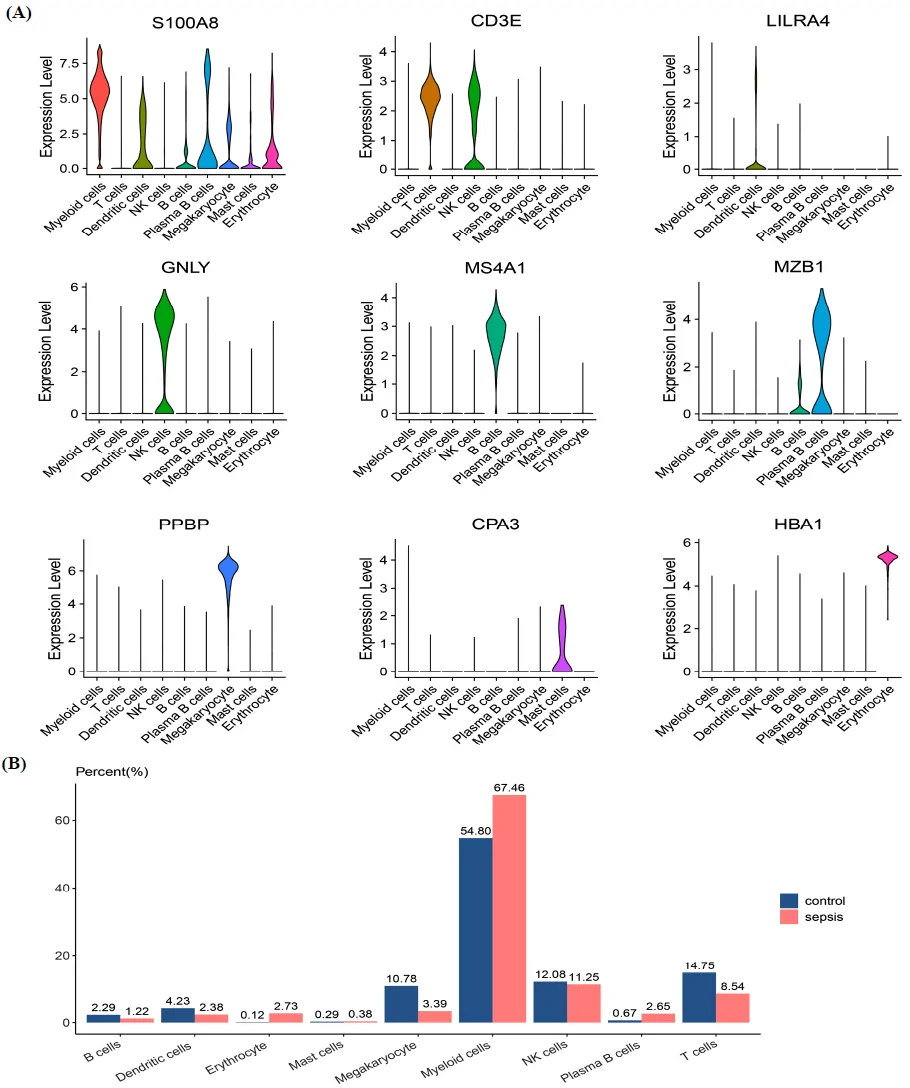
Cell difference analysis among various samples. (**A**) Violin plot of marker gene expression in different cell clusters (X axis is the cell subtypes, and the Y axis is the expression of marker genes). (**B**) The cell proportion difference among various sample groups (X axis is the cell subtypes and Y axis is the percentage).

After cell filtration, SCT standardization and unsupervised cell clustering, we eventually obtained 9 cell subgroups from 41,322 cells, including myeloid cells, T cells, dendritic cells, NK T cells, B cells, plasma B cells, megakaryocytes, mast cells and erythrocytes (Fig. **1A** and **B**). The number of cells in each cell subpopulation is shown in Supplementary Fig. (**1**). Gene numbers in each sample ranged from 200 to 6000 (Fig. **1C**), and in each cell, they were between 200 and 2000 (Fig. **1D**), with mitochondrial genes less than 10% (Fig. **1E**). The marker genes of the cell cluster were shown in the bubble plot (Fig. **1F**). We observed that *S100A9* and *S100A8* were overexpressed in myeloid cells, while *CCR7* and *CD3E* were overexpressed in T cells (Fig. **2A**). Additionally, we analyzed the cell proportions in both the normal and sepsis groups. We found that the proportions of myeloid cells, erythrocytes, and mast cells were higher in the sepsis group (Fig. **2B**).

### 3.2 Activated p53 and IL-17 of Myeloid Cells were Crucial for Sepsis Progression

**Fig. (3):**
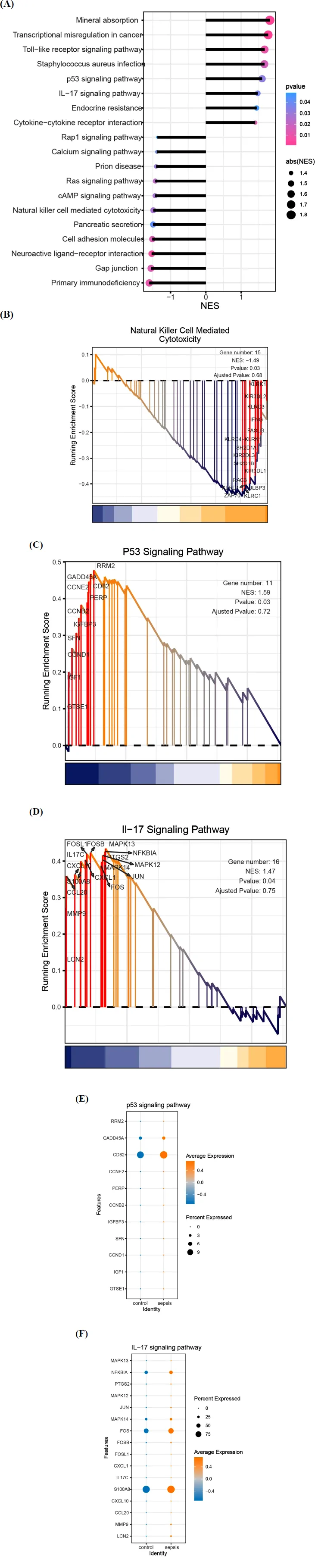
Functional enrichment difference of Myeloid cells. (**A**) KEGG enrichment analysis of Myeloid cells in sepsis and normal groups. (**B**) The significantly upregulated genes in the Natural Killer Cell-Mediated Cytotoxicity pathway. (**C**) The significantly up-regulated genes in the p53 signaling pathway. (**D**) The significantly up-regulated genes in the IL-17 signaling pathway. (**E**) Bubble plot of gene expression of the P53 signaling pathway in the normal and sepsis groups. (**F**) Bubble plot of gene expression of the IL-17 signaling pathway in the normal and sepsis groups.

We identified the DEGs of myeloid cells in the normal and sepsis groups. The GO biological process and KEGG enrichment analyses showed that the inflammation and signal transduction pathways, such as transcriptional misregulation in cancer, the Toll-like receptor signaling pathway, Staphylococcus aureus infection and the p53 and IL-17 signaling pathways, were significantly enriched in the sepsis group. In contrast, the signal transduction pathways, such as Gap junction, neuroactive ligand-receptor interaction and natural killer cell-mediated cytotoxicity, were significantly enriched in the normal group (Fig. **3A**). Specifically, KLRK1, KIR3DL2, IFNG, and KLRC3, involved in the natural killer cell-mediated cytotoxicity (Fig. **3B**); GADD45A, CD82, CCNE2, and SFN, involved in the p53 signaling pathway (Fig.**3C**); and MMP9, FOS, LCN2, and S100A8, involved in the IL-17 signaling pathway (Fig. **3D**), were significantly upregulated in the sepsis group. The marker genes of the p53 and IL-17 signaling pathways (Figs. **3E** and **F**) were overexpressed in the sepsis groups, indicating that these pathways played a crucial role in sepsis progression.

### 3.3 Macrophages, Megakaryocytes and Neutrophils may be Key Contributors to Anti-infection Activation in Sepsis

**Fig. (4):**
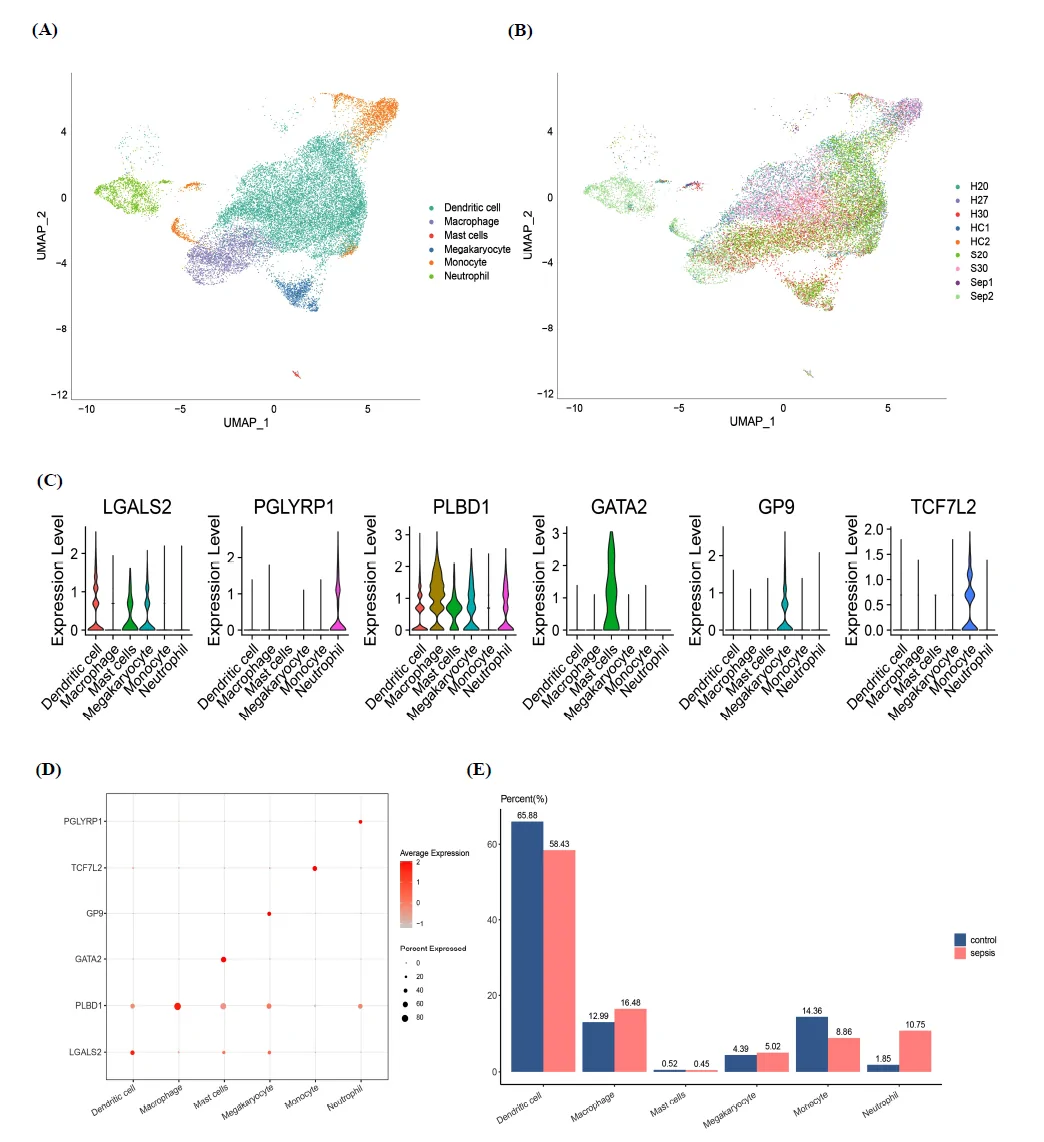
The heterogeneity analysis of Myeloid cells. (**A**) UMAP of plot Myeloid cells sub-clustering. (**B**) UMAP plot of the Myeloid cell subtype distribution in different samples. (**C**) The expression of marker genes in different cell subtypes. (**D**) Bubble plot of marker gene expression. (**E**) The proportion of cells of the cell subtype in the normal and sepsis groups.

To further elucidate the heterogeneity within myeloid cells, we subdivided them into dendritic cells, macrophages, mast cells, megakaryocytes, monocytes and neutrophils (Fig. **4A** and **B**), and the marker genes of each cell were shown in (Fig. **4C** and **D**). For instance, *PGLYRP1* was significantly overexpressed in neutrophils, while *GATA2* was significantly expressed in mast cells. Notably, the proportions of macrophages, megakaryocytes, and neutrophils were significantly increased in the sepsis group compared to the normal controls (Fig. **4E**), indicating that these cells may be key immune cells recruited for anti-infection activation in sepsis.

#### 3.3.1 JUNB and SPI1 were two crucial TFs affecting multiple immune pathways

**Fig. (5):**
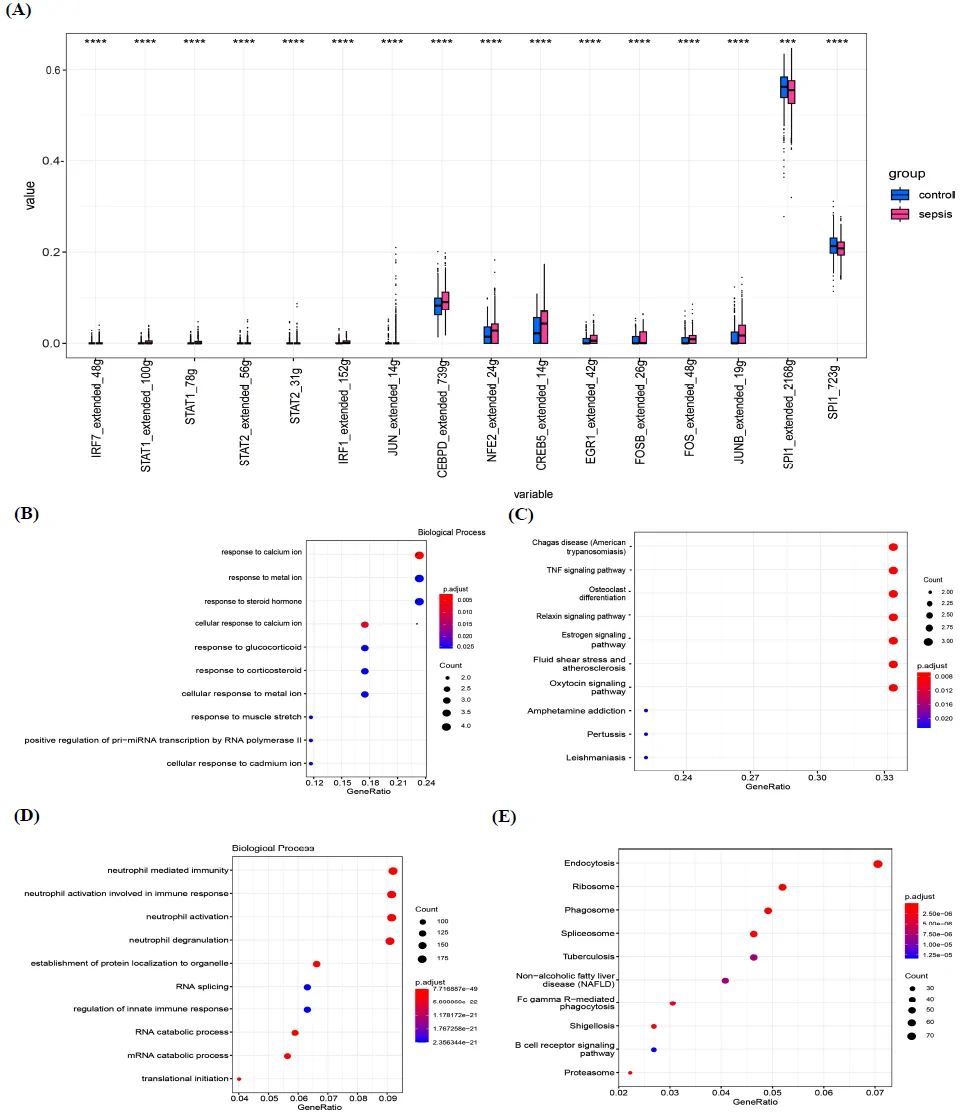
Identifying the Transcription Factors (TFs) of Myeloid cells. (**A**) The difference of key TFs in normal and sepsis groups. (**B**) The biological process of JUNB-mediated network. (**C**) The KEGG enrichment pathway of the JUNB-mediated network. (**D**) The biological process of SPI1-mediated network. (**E**) The KEGG enrichment pathway of the SPI1-mediated network.

The SCENIC and AUCell algorithms were used to identify key regulons in myeloid cells. We compared the differences in these regulons between the normal and sepsis groups. We found that a series of key regulons were significantly upregulated in the sepsis group, such as STAT1_78g, STAT2_31g, CEBPD_extended_739g, NFE2_extended_14g, JUNB_extended_19g, and SPI1_extended_2168g (Fig. **5A**). Notably, *JUNB* mediated the biological processes of calcium ion, steroid hormone, and glucocorticoid response activation (Fig. **5B**) and pathways, including the TNF signaling pathway, osteoclast differentiation, the Relaxin signaling pathway, and the estrogen signaling pathway, (Fig. **5C**), which indicated that these pathways were crucially involved in sepsis. In addition, *SPI1* was involved in the biological process of neutrophil-mediated immunity, neutrophil activation involved in immune response, neutrophil degranulation activation (Fig. **5D**) and mediated endocytosis, Ribosome, phagosome, Fc gamma R-mediated phagocytosis pathways (Fig. **5E**), suggesting that *SPI1* was another key hub supporting the anti-infection response in sepsis.

### 3.4 MPZL1, FAS and L1CAM Mediated Cell Communication in Sepsis

**Fig. (6):**
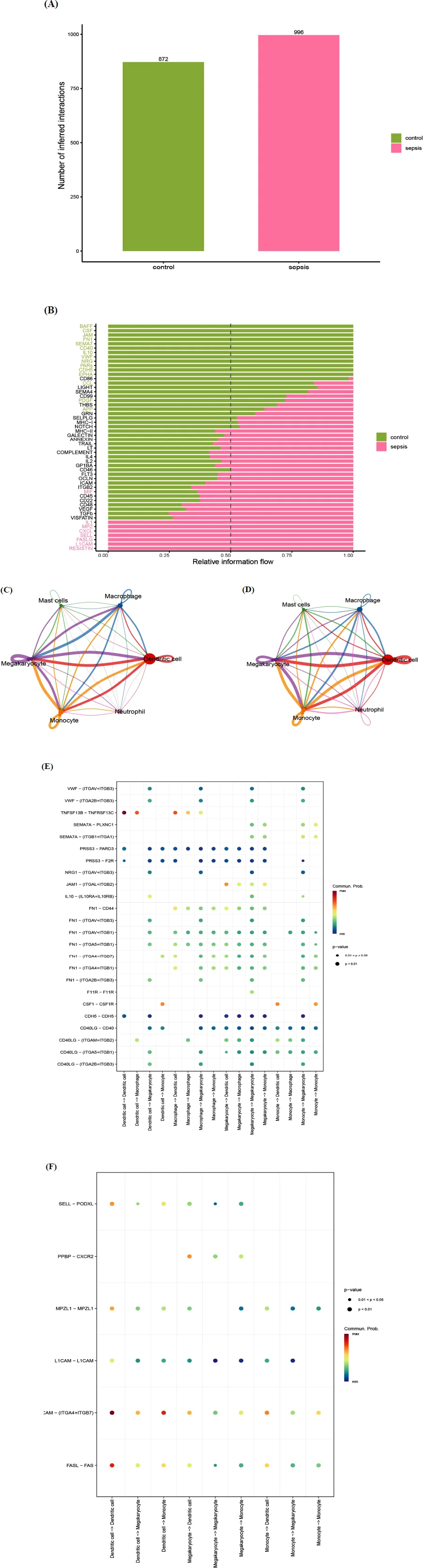
Cell-cell communication among Myeloid cell subtypes. (**A**) Comparison of the number of cell communications in Myeloid cells in the normal and sepsis group. (**B**) Several signaling components were analyzed in both normal and sepsis groups. (**C**) The cell interaction network in the normal group. (**D**) The cell interaction network in the sepsis group. (**E**) The cell interaction way analysis in the normal group. (**F**) Cell interaction analysis in the sepsis group.

Cell-cell communication analysis revealed that cells in sepsis had higher communication strength (Fig. **6A**), and that some cytokines or signal components, such as RESISTIN, L1CAM, FASLG, CXCL and IL-1, were more likely to be produced in the sepsis group (Fig. **6B**). In the normal group of cell interaction network, megakaryocytes, monocytes, and dendritic cells were key interaction hubs as the senders and responders of various signaling pathways (Fig. **6C**), and they were also key hub genes with more complex interactive networks (Fig. **6D**). Furthermore, the PRSS3-PARD3, PRSS3-F2R, FN1-(ITGAV+ITGB1), FN1-(ITGA5+ITGB1) and FN1-(ITGA4+ITGB7) were the key receptor-ligand pairs in the normal group (Fig. **6E**), but the SELL-PODXL, MPZL1-MPZL1, L1CAM-L1CAM, L1CAM-(ITGA4+ITGB7) and FASL-FAS were the main receptor-ligand pairs in the sepsis group (Fig. **6F**). These findings suggest that these key points could be potential therapeutic targets for sepsis.

## 4 DISCUSSION

Sepsis is a life-threatening heterogeneous symptom that will lead to acute organ dysfunction and septic shock, showing an increasing trend of incidence and death rate. Similar to acute myocardial infarction, polytrauma or stroke, the speed and effectiveness of therapy administered in the initial stage of sepsis are likely to influence the final outcome. Although the pathophysiology of sepsis is not fully understood, it is clear that sepsis involves a wide range of interactions between pathogens and the immune system [22]. Therefore, exploring the characteristics of these circulating immune cells is crucial in understanding the pathogenesis and developing more effective intervention strategies for the treatment and prevention of sepsis [23].

This study utilized scRNA-seq analysis to explore the specific immune activity of the circulating myeloid cells in sepsis. Similar to previous studies, sepsis exhibits a dual-phase immune activation, including both anti-inflammatory and pro-inflammatory immune responses against pathogens [24], which profoundly perturbs immune homeostasis. In our analysis, myeloid cells exhibited pronounced pro-inflammatory activation of the IL-17 and p53 signaling pathways. Notably, the sustained overexpression of IL-17 pathway markers implies persistent recruitment and hyperactivation of neutrophils and macrophages, which perpetuate the inflammatory cascade, augment tissue damage, and contribute to the development of a cytokine storm in severe cases [25, 26]. Concurrently, continuous activation of p53-associated markers reflects prolonged cellular stress and DNA damage response, which not only induce apoptosis and cell-cycle arrest in immune cells but also compromise their functional adaptability, thereby amplifying immune dysregulation [27, 28]. Furthermore, myeloid cells were subdivided into macrophages, megakaryocytes, and neutrophils, all of which were expanded in sepsis and contributed to the excessive release of inflammatory cytokines, chemokines, proteases, and reactive oxygen species [29] through specific gene regulatory networks and intercellular communication axes. Although other studies have reported challenges in clearly distinguishing between immunosuppressive and pro-inflammatory phases at the transcriptomic level in sepsis [30], our findings suggest that focusing on pivotal regulatory pathways, such as IL-17 and p53, provides valuable mechanistic insight into the pathological role of myeloid cells in sepsis-associated immune dysregulation. Sepsis can be classified into an initial hyperinflammatory phase characterized by SIRS and the subsequent immunosuppressive phase characterized by organ dysfunction with hypoinflammatory features (exhaustion and death of myeloid and lymphoid lineages) [31]. Inflammation generally refers to a host protective response induced by invading pathogens or endogenous signals, which can be sensed by immune receptors on myeloid cells, including neutrophils, macrophages, monocytes and Dendritic Cells [32]. These immune cells secrete substantial quantities of proinflammatory cytokines, such as IL-1β, IL-12, IL-18, and tumor necrosis factor-α (TNF-α), to mediate a wider immune response, leading to acute protein synthesis in the liver, fever, and platelet activation [33]. Notably, cell debris and alarmins produced by damaged cells can amplify receptor activation, further inducing hyperinflammation [34]. Blocking inflammatory cytokines protects against acute sepsis in an animal model [35]. IL-17 and p53 signaling pathways are significantly activated in the myeloid cells of sepsis, suggesting that blocking these pathways may produce a more effective anti-inflammatory effect during sepsis treatment.

In addition, JUN B proto-oncogene (JUNB) and SPI1 are two identified TFs that underpin the regulation of inflammation response. *JUNB* has been reported to play an important regulatory role in innate and adaptive immunity by regulating cytokine secretion, differentiation, and maturation of immune or myeloid cells, such as macrophages, T cells, and dendritic cells, and enhancing the effector functions of natural killer cells and neutrophils [36]. Notably, *JUNB* promotes the transcription of IL-23R and IL-17A genes during CD4^+^ T cell differentiation, participating in immune system disorders and autoimmune diseases [37], while also promoting the infiltration of neutrophils and macrophages [38]. From a therapeutic perspective, targeting *JUNB* may offer novel opportunities for modulating excessive inflammatory responses in sepsis. For instance, strategies such as RNA interference approaches directed against *JUNB* could theoretically dampen downstream pro-inflammatory signaling cascades, thereby reducing cytokine release and immune cell overactivation [36, 39]. Although these strategies remain at an experimental stage, they highlight the potential of *JUNB* as a promising therapeutic target in the management of sepsis. *SPI1* is another key identified TF in sepsis and plays a pivotal role in myeloid and lymphoid lineage development [40]. Knockdown of *SPI1* reduced the production of proinflammatory cytokines in sepsis [41]. Xie *et al.* reported that SPI1 is highly expressed in sepsis and enhances monocyte autophagy by regulating ANXA1 [42]. Overall, the two TFs may contribute to the polarization fate and immune function of myeloid cells in sepsis. Their interaction with receptor genes (*MPZL1, FAS*, and *L1CAM*) mediates crucial cellular communication networks. Hence, targeting these TFs and their associated receptors may be expected to serve as potential therapeutic targets for the development of effective anti-inflammatory drugs in sepsis treatment.

## CONCLUSION

In general, sepsis is regarded as a biphasic disease with an initial hyperinflammatory phase and a late immunosuppression phase. Multiple novel therapies have been developed to combat sepsis, for instance, resuscitative strategies, antibiotic therapy and ventilator use; however, the failure of anti-inflammatory drug trials hinders our understanding of sepsis immunopathology. In this study, we revealed the immune features of myeloid cells and their key transcriptional regulation using scRNA-seq analysis of sepsis samples. Our findings are expected to elucidate the underlying pro-inflammatory mechanisms of immune cells in sepsis and improve future therapeutic tools.

## LIMITATIONS

Although this study provided valuable insights, several limitations still existed. Firstly, our sample size was small (4 sepsis patients and 5 controls), which may limit the broader applicability of the findings. Secondly, the identified pathways and targets require experimental validation at the functional or protein level. Thirdly, the dataset may not fully represent the heterogeneity of sepsis and patient variability, which could influence the interpretation of immune cells. Lastly, large-scale single-cell data collected from different stages of sepsis need to be analyzed to better understand the dynamic immune changes in sepsis. Finally, further studies with larger cohorts and experimental validations are also required.

## Data Availability

All data generated or analyzed during this study are included in this published article.

## LIST OF ABBREVIATIONS

IL-17: Interleukin-17
JUNB: JUN B Proto-oncogene
ScRNA-Seq: Single Cell RNA Sequencing
TNF-α: Tumor Necrosis Factor-α
GEO: Gene Expression Omnibus
PCA: Principal Component Analysis
GSEA: Gene Set Enrichment Analysis
BP: Biological Process
GO: Gene Ontology
KEGG: Kyoto Encyclopedia of Genes and Genomes
DEGs: Differentially Expressed Genes
GRNs: Gene Regulatory Networks
TFs: Transcription Factors
