# Supplementary material for: Single-Cell Profiling Identifies JUNB/SPI1-Driven Inflammatory Programs and Novel Communication Axes in Myeloid Cells of Sepsis
Source: Endocr Metab Immune Disord Drug Targets. 2025 Oct 16;26:E18715303425071. doi: 10.2174/0118715303425071250925075308 (PMC13523166; doi:10.2174/0118715303425071250925075308)
Supplement: Supplementary file 1 [file EMIDDT-26-E18715303425071_SD1.pdf]

Supplementary Material

Single-Cell Profiling Identifies JUNB/SPI1-Driven Inflammatory Programs and Novel Communication Axes in Myeloid Cells of Sepsis

Liyao Liu[1], Lin Zhao[1,*] and Jixiang Tan[1,*]

[1]*Department of Emergency, The First Affiliated Hospital of Chongqing Medical University, Chongqing, 400016, China*

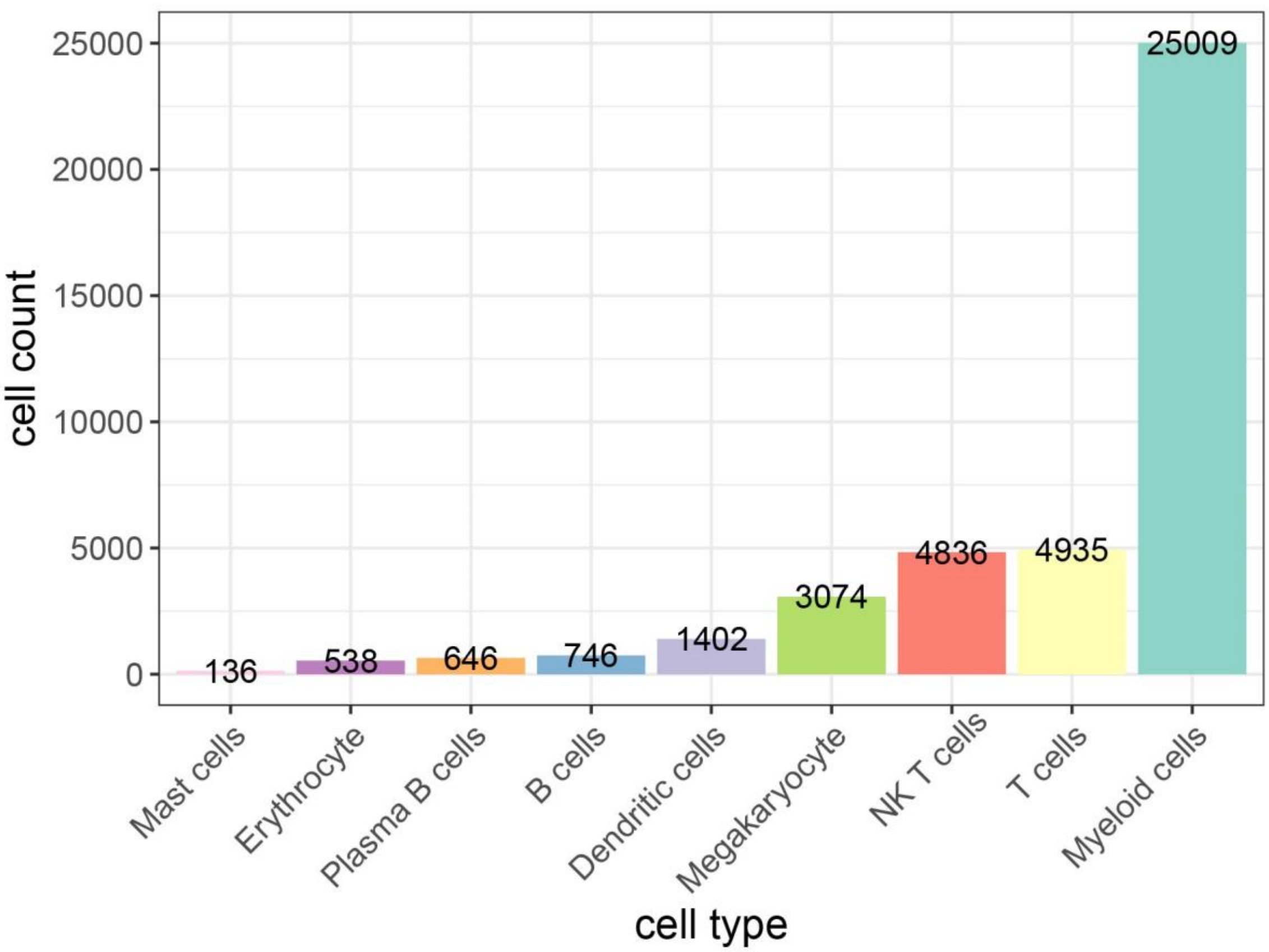

Fig. (S1). The display of the number of cells in each of the nine cell subpopulations (myeloid cells, T cells, dendritic cells, NK T cells, B cells, plasma B cells, megakaryocytes, mast cells, and erythrocytes) identified in sepsis.
